# One hundred research questions in conservation physiology for generating actionable evidence to inform conservation policy and practice

**DOI:** 10.1093/conphys/coab009

**Published:** 2021-04-07

**Authors:** Steven J Cooke, Jordanna N Bergman, Christine L Madliger, Rebecca L Cramp, John Beardall, Gary Burness, Timothy D Clark, Ben Dantzer, Erick de la Barrera, Nann A Fangue, Craig E Franklin, Andrea Fuller, Lucy A Hawkes, Kevin R Hultine, Kathleen E Hunt, Oliver P Love, Heath A MacMillan, John W Mandelman, Felix C Mark, Lynn B Martin, Amy E M Newman, Adrienne B Nicotra, Graham D Raby, Sharon A Robinson, Yan Ropert-Coudert, Jodie L Rummer, Frank Seebacher, Anne E Todgham, Sean Tomlinson, Steven L Chown

**Affiliations:** 1 Fish Ecology and Conservation Physiology Laboratory, Department of Biology and Institute of Environmental and Interdisciplinary Science, Carleton University, 1125 Colonel By Dr., Ottawa, Ontario K1S 5B6, Canada; 2 School of Biological Sciences, The University of Queensland, Brisbane 4072, Australia; 3 Securing Antarctica’s Environmental Future, School of Biological Sciences, Monash University, Clayton, Victoria 3800, Australia; 4 Department of Biology, Trent University, 1600 West Bank Drive, Peterborough, Ontario K9L 0G2, Canada; 5 School of Life and Environmental Sciences, Deakin University, Geelong, Victoria 3216, Australia; 6 Department of Psychology, Department of Ecology & Evolutionary Biology, Ann Arbor, MI 48109, USA; 7 Instituto de Investigaciones en Ecosistemas y Sustentabilidad, Universidad Nacional Autónoma de México, Antigua Carretera a Pátzcuaro 8701, Morelia, Michoacán, 58190, Mexico; 8 Department of Wildlife, Fish & Conservation Biology, University of California, Davis, One Shields Avenue, Davis, CA 95616, USA; 9 Brain Function Research Group, School of Physiology, University of the Witwatersrand, 7 York Rd, Parktown, 2193, South Africa; 10 College of Life and Environmental Sciences, Hatherly Laboratories, University of Exeter, Prince of Wales Road, Exeter EX4 4PS, UK; 11 Department of Research, Conservation and Collections, Desert Botanical Garden, Phoenix, AZ 85008, USA; 12 Smithsonian-Mason School of Conservation, 1500 Remount Road, Front Royal, VA 22630, USA; 13 Department of Integrative Biology, University of Windsor, 401 Sunset Avenue, Windsor, Ontario N9B 3P4, Canada; 14 Department of Biology and Institute of Biochemistry, Carleton University, 1125 Colonel By Dr., Ottawa, Ontario K1S 5B6, Canada; 15 Anderson Cabot Center for Ocean Life, New England Aquarium, 1 Central Wharf, Boston, MA, 02110, USA; 16 Department of Integrative Ecophysiology, Alfred Wegener Institute Helmholtz Center for Polar and Marine Research, Am Handelshafen 12, 27570 Bremerhaven, Germany; 17 Global Health and Infectious Disease Research, University of South Florida, 3720 Spectrum Boulevard, Tampa, FL 33612, USA; 18 Department of Integrative Biology, University of Guelph, Guelph, Ontario N1G 2W1, Canada; 19 Research School of Biology, Australian National University, Canberra, Australian Capital Territory 2601, Australia; 20 School of Earth, Atmospheric and Life Sciences (SEALS) and Centre for Sustainable Ecosystem Solutions, University of Wollongong, Wollongong, New South Wales 2522, Australia; 21 Centre d'Etudes Biologiques de Chizé, CNRS UMR 7372—La Rochelle Université, 79360 Villiers-en-Bois, France; 22 ARC Centre of Excellence for Coral Reef Studies, James Cook University, Townsville, Queensland 4811, Australia; 23 School of Life and Environmental Sciences A08, University of Sydney, New South Wales 2006, Australia; 24 Department of Animal Science, University of California Davis, Davis, CA 95616, USA; 25 School of Biological Sciences, University of Adelaide, North Terrace, Adelaide, South Australia 5000, Australia

**Keywords:** Biodiversity threats, conservation decisions, conservation physiology, evidence, research questions

## Abstract

Environmental change and biodiversity loss are but two of the complex challenges facing conservation practitioners and policy makers. Relevant and robust scientific knowledge is critical for providing decision-makers with the actionable evidence needed to inform conservation decisions. In the Anthropocene, science that leads to meaningful improvements in biodiversity conservation, restoration and management is desperately needed. Conservation Physiology has emerged as a discipline that is well-positioned to identify the mechanisms underpinning population declines, predict responses to environmental change and test different *in situ* and *ex situ* conservation interventions for diverse taxa and ecosystems. Here we present a consensus list of 10 priority research themes. Within each theme we identify specific research questions (100 in total), answers to which will address conservation problems and should improve the management of biological resources. The themes frame a set of research questions related to the following: (i) adaptation and phenotypic plasticity; (ii) human–induced environmental change; (iii) human–wildlife interactions; (iv) invasive species; (v) methods, biomarkers and monitoring; (vi) policy, engagement and communication; (vii) pollution; (viii) restoration actions; (ix) threatened species; and (x) urban systems. The themes and questions will hopefully guide and inspire researchers while also helping to demonstrate to practitioners and policy makers the many ways in which physiology can help to support their decisions.

## Teaser

Conservation physiology has emerged as an integrative action-oriented discipline. Yet, there remain many ways in which conservation physiology could further contribute to addressing the biodiversity crisis. We share a list of 100 research questions related to conservation physiology that, if answered, would enhance the conservation of biodiversity.

## Introduction

Humans have become such a dominant agent in global ecosystems that we have now entered the ‘Anthropocene’—a distinct geological epoch where human activity has a dominant influence on climate and the environment (Vitousek *et al.,* 1997; Crutzen, 2006). An unfortunate element of that dominance is a biodiversity crisis so extreme as to have precipitated the sixth major extinction in Earth’s history (Chapin *et al.,* 2000). Nevertheless, many in the biodiversity conservation movement still find cause for optimism (Balmford and Knowlton, 2017), because relevant and robust evidence can still be the basis for success in conservation, restoration and management of biodiversity, wildlife populations and ecosystems in ways that benefit nature and humans (Sutherland *et al.,* 2004; Rose *et al.,* 2018). Efforts to identify the broadly relevant research questions that, if addressed, have great potential to improve conservation policy and practice and have become a popular strategy to ensure that scientific efforts are focused appropriately (Sutherland *et al.,* 2006, 2009). Such efforts have targeted different issues (e.g. agriculture, Pretty *et al.,* 2010), realms (e.g. marine systems, Parsons *et al.,* 2014), regions (e.g. Canada, Rudd *et al.,* 2011; Antarctica, Chown *et al.,* 2015) and, most recently, sub-disciplines (e.g. conservation behaviour, Greggor *et al.,* 2016) and taxonomic groups (e.g. seed biology, Saatkamp *et al.,* 2019).

Conservation physiology has emerged as a novel sub-discipline of conservation science focused on the use of physiological knowledge, concepts and tools to identify and solve conservation problems (Wikelski and Cooke, 2006; Cooke *et al.,* 2013). The utility of conservation physiology lies in its ability to reveal cause-and-effect relationships (Cooke and O’Connor, 2010), which in turn allow predictions to be made of how organisms, populations and ecosystems will respond to environmental change (Seebacher and Franklin, 2012). As this area of study matures and evolves (Cooke *et al.,* 2020), more success stories in conservation physiology are becoming apparent (Madliger *et al.,* 2016; Madliger *et al.,* 2021). For example, physiological research is increasingly being used to inform endangered species recovery planning (Birnie-Gauvin *et al.,* 2017; Mahoney *et al.,* 2018). Nevertheless, a physiological paradigm can still engage and be engaged more broadly with conservation science to optimize practical and policy outcomes. One of the challenges with conservation physiology is that, because it is a nascent discipline, its relevance is not always obvious or understood by conservation practitioners and decision-makers. To that end, we present here a collaboratively derived science–policy–practice research agenda (Sutherland *et al.,* 2012) focused on developing and clarifying the evidence in support of conservation physiology (Milner-Gulland *et al.,* 2010). Specifically, we generated a list of 10 priority areas in which conservation physiology has potential for helping to address conservation problems and improving the management of biological resources. Within each theme we have identified key research questions using the methods outlined in Sutherland *et al.* (2011). The team that generated the 10 priority areas and 100 research questions included diverse conservation physiology experts, as well as established and emerging leaders from 8 countries whose research spans all continents and relevant taxa.

## Methods

### Participants

We recruited 28 experts in conservation physiology—most of them representatives from the Editorial Board of the journal *Conservation Physiology*—to participate in formulating important and unanswered research questions. Editorial Board members are inherently recruited to span diversity (of all forms) and because of their expertise. Moreover, editors are privileged in that they have access to frontier science and thus are strategically positioned to be both reflective and forward-looking in their perspectives. Several additional early career researchers active in the conservation physiology community also participated in an effort to increase the diversity of perspectives represented. The experts spanned metazoan taxa (e.g. from plants to animals), ecosystems (e.g. from the oceans to alpine ecosystems) and regions (i.e. contributors are based in eight countries including Australia, Canada, France, Germany, Mexico, South Africa, the United Kingdom and the United States; however, conduct research from pole to pole). Contributors are listed as co-authors and participated in several rounds of question editing, development of the final list of questions below and writing of the manuscript. Various researchers and research groups worldwide are already attempting to tackle some of the questions listed here. Our team of authors (especially those on the Editorial Board) have been inspired by those ongoing research efforts and are humbled by our ability to amass those ideas, organize them and pass this inspiration on.

**Figure 1:**
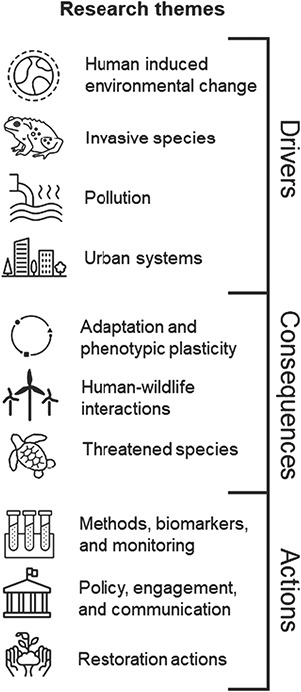
Visualization of the research themes that include the 100 questions related to conservation physiology generated in this paper. The themes broadly cover the drivers of conservation issues, their consequences and actions to address conservation issues or otherwise advance the impact of conservation physiology.

### Preliminary formulation of questions

We requested each contributor to independently provide up to 10 research questions. To be considered, questions had to meet the following criteria: were not overly specific (e.g. effects of pollutant X on species Y), were answerable through a reasonable research design and were related to physiology and mechanism. In total, 245 questions were submitted at the first stage (see Supplementary Information) equating to approximately 9 questions from each author (recognizing some provided more and some provided fewer).

### Thematizing questions

Each question was individually screened and classified into major thematic areas that emerged from clustering questions into logical categories (Fig. 1). A total of 10 themes emerged: (i) adaptation and phenotypic plasticity; (ii) human-induced environmental change; (iii) human–wildlife interactions; (iv) invasive species; (v) methods, biomarkers and monitoring; (vi) policy, engagement and communication; (vii) pollution; (viii) restoration actions; (ix) threatened species; and (x) urban systems. We acknowledge that these themes are subjective and that some questions may be relevant to several themes. The questions presented here emerged iteratively based on interactions among the authors. Once categorized, theme-specific questions were sent for review to an author who is an expert in that field. Experts were asked to screen all questions for relevance and then subsequently approve, reject or revise. Duplicate and highly similar questions were collated. Once the list of theme-specific questions was returned, all questions were collated into a semi-final list for consideration by the entire expert panel.

### Final list of questions

The semi-final list of questions was posted on an open forum for all authors to contribute their comments, edits and thoughts. Authors were asked to carefully review each question and approve, reject or revise. Authors were given 2 weeks to provide edits at which point the document was closed and downloaded for final revisions. Questions were modified to address any key concerns and collated into the final list of questions provided in this paper. This list of 100 questions is not prioritized (i.e. question 1 is no more or less important than question 100), but each question has received careful thought and refinement. Although it is possible to use additional expert-ranking processes to prioritize questions, the reality is that priorities will vary among regions, ecosystems, issues, actors and taxa/species. Themes are ordered alphabetically in an attempt to not prioritize one theme over another. We also acknowledge that some questions could easily fit within multiple themes, which is an inherent reality of these cross-cutting questions and themes. The questions presented here were collected prior to the COVID-19 pandemic and we opted to not tailor any questions specific to the pandemic; a short list of research questions specific to COVID-19 and conservation physiology are presented separately in Cooke *et al.* (2021).

## Themes and research questions

### Adaptation and phenotypic plasticity

Conservation actions rely, either implicitly or explicitly, on projections about how threatened populations will fare in the near and long term with and without those actions. Thus, some of the biggest questions in biology focus on uncertainty about the pace of adaptation and the potential for phenotypic plasticity to affect conservation outcomes. Phenotypic plasticity describes the range of phenotypes that a given genotype can express in response to environmental variation; plasticity encompasses the related concepts of acclimation and flexibility, which may involve short- or long-term adjustments to physiology. Quantifying the extent of genetic and phenotypic variation in physiological responses is one way to inform projections for organisms threatened by a rapidly changing environment (Padfield *et al.,* 2016). Knowing (i) the plasticity of physiological traits, (ii) the potential for transgenerational effects and (iii) the potential for rapid adaptation of physiological tolerance or plasticity can be crucial to projections about how a given species will fare (Seebacher *et al.,* 2015, Veilleux *et al.,* 2015). Furthermore, there is also a need to understand the cause-and-effect links between genes, physiological phenotypes and environmental conditions, in part because those links can explain how genomic data can inform conservation decisions. A key example here are thermal performance curves, which are often based on physiological performance, and are routinely used to assess the prospects of species under future warming scenarios (Pörtner and Farrell, 2008; Schulte, 2015; Sinclair *et al.,* 2016; Deutsch *et al.,* 2020). Performance curves are often presented at the species-level but contain inter-individual variation, the material of natural selection (Roche *et al.,* 2016). Individual animals can vary in the shape (e.g. height and breadth) of their curves due to inter-individual variation, life stage or as the organism acclimates to changes in its environment and the mechanisms underlying these various changes may be shared or mutually exclusive. To generate usable knowledge, it is necessary to assess components of phenotypic plasticity and genetic variation in physiological traits using ecologically relevant measurement conditions and reliable indicators of performance. Thus, the questions in this theme are some of the most challenging ones faced by comparative physiologists, both within and beyond the realm of conservation.

1. Can the potential for rapid evolution in physiological tolerance of threatened taxa be maximized?
2. Does the evolutionary history of a species determine how underlying physiological mechanisms maximize performance and fitness under environmental change?
3. How much does individual variation in physiological plasticity contribute to resilience to change in a conservation framework?
4. Is there a link between phenotypic plasticity and generation time (to buffer slow rates of evolution)?
5. How important is within-individual physiological flexibility when examining the responses of a group of organisms to a stressor?
6. How do interactions among plasticity, genetic drift and adaptation affect the resilience of populations to environmental change?
7. How do symbionts (e.g. gut microbiome, root symbionts) respond to a modified environment and how does it affect fitness?
8. Which physiological traits are best/most appropriate for informing whether wildlife harvest practices (i.e. fisheries) exert selective pressures?
9. What physiological mechanisms determine the pace of thermal acclimation and adaptation?

### Human–induced environmental change

Contemporary organisms are challenged with natural environmental variability and disturbances and anthropogenic changes. Several of the leading anthropogenic stressors that alter local environments have indeed been listed throughout this article (e.g. invasive species, pollution), though habitat fragmentation and degradation, pathogen transport and the emergence of new pathogens and dramatic increases in greenhouse gas emissions have significantly impacted our world as well (Mittermeier *et al.,* 2011). To effectively preserve and restore our remaining biodiversity, scientists typically argue for a proactive approach to conservation—in contrast to reactive—whereby management actions are executed before demographic instability or extinction risk occurs and are usually more cost- and time-effective (Drechsler *et al.,* 2011; Sterrett *et al.,* 2019). Because an individual’s physiology can rapidly changes to reflect disturbance, relevant physiological parameters could be monitored and/or evaluated to determine the severity of environmental stressors on organisms (Bergman *et al.,* 2019) and could therefore be used to prevent population collapse and/or extinction (i.e. to identify tipping points; Dai *et al.,* 2012). As global environments continue to transform with climate and anthropogenic change, it will be vital to understand the physiological capacity of organisms to predict future species distributions and population dynamics for the development of effective conservation strategies (Williams *et al.,* 2008; Fuller *et al.,* 2010).

How is immune function and disease susceptibility influenced by anthropogenic change?
Are there physiological and genetic interventions that can be exploited (e.g. through selective breeding, translocation) for species at risk of climate-induced extirpation/extinction?
How can physiological metrics (e.g. tree growth ring analysis, metabolic rate measures in ectotherms) provide long-term predictions of organismal sensitivity to global change?
How can data on physiological traits and adaptive capacity be used to identify potential ‘winners’ and ‘losers’ in a conservation triage process, and which traits best fit this application?
What are the connections between physiological research and the economic consequences of climate change, and how can these connections be best identified and communicated to improve conservation in the face of rapid climate change?
How do changes in winter climate and snow and/or ice cover influence overwintering physiology of plants and animals?
How can the complexity of natural environments and interacting stressors be better incorporated into conservation physiology research?
How will physiological systems adapt and respond to the interactive and cumulative effects of climate change?
To what extent can evolutionary rescue (trait evolution over ecological timescales) mitigate the long-term effects of global climate change on species of concern (both in context of proactive management and natural selection)?
To what extent does physiological resilience (or lack thereof) to environmental change of one target species affect or predict success of another?
What infrastructure is needed (e.g. reciprocal common gardens, phenotyping centres, whole-genome sequencing, remote sensing methodologies) to enhance research on plant responses to climate change and inform restoration actions?
What physiology underpins local and traditional (i.e. Indigenous rightsholders) knowledge about species’ resilience or sensitivity to changing environments?
How can physiological tools be best used to improve our capacity to monitor organismal and population responses to environmental change?
How important are carry-over effects, plasticity and epigenetic modifications in improving resilience of organisms to environmental change?
What are the physiological responses at different levels of organization—individual, population, species, assemblage—and how do they influence community responses to anthropogenic change?

### Human–wildlife interactions

Human–wildlife interactions are increasing globally as a consequence of human population growth and land-use changes (Madden, 2004; White and Ward, 2011). Human–wildlife interactions are often framed as conflicts (Peterson *et al.,* 2010) and can include injury or death of humans and non-human animals (e.g. livestock and damage to crops or other human possessions) (Richardson *et al.,* 2020). This type of wildlife conflict has captured the attention of practitioners, resource managers and researchers and is considered to be one of the most critical challenges facing wildlife conservation (Frank *et al.,* 2019). Wild animals too face death or injury as a result of human–wildlife interactions, both directly (e.g. wildlife-vehicle collisions, culling as a management strategy) and indirectly (e.g. fences, dams, habitat fragmentation, etc.). Protected areas are one solution that can provide refuge for wildlife away from human activity and have thus been considered ‘habitat(s) surrounded by seas of cultivation and development’ (Madden, 2004). Although physiology is inherently linked with how animals respond to their environments, the use of physiological information to manage human–wildlife interactions has been limited in this context (though see Blackwell and Fernandez-Juricic, 2013). Physiological approaches hold the potential to prevent conflict from occurring entirely or to reduce the frequency or severity of the conflict (Madliger *et al.,* 2018; Elmer *et al.,* 2021).

Can physiological knowledge be used to mitigate predator-related human–wildlife conflict?
How can sensory physiology be manipulated to guide animals away from, and prevent collisions with, human infrastructure (e.g. roads, windows, turbines) and towards safe passage (e.g. wildlife under/overpasses, fishways)?
How does human infrastructure and operations (e.g. hydropower, wind turbines, roads) affect the physiological status of wild organisms?
How does human presence (e.g. ecotourism) affect the physiology and welfare of animals?
Can physiological metrics/tools be incorporated into the economic valuation of ecosystems for conservation and protection?
What physiological tools and concepts can be used to inform the siting, design and management of protected areas?
What are the physiological consequences of supplemental feeding (intended—e.g. bird feeders; unintended—e.g. garbage) for wild flora and fauna?

### Invasive species

Biological invasions are a pervasive consequence of an increasingly connected planet and expanding human population (Pyšek *et al.,* 2020). After habitat loss, invasive species—species that spread widely in regions where they are not native and adversely impact local wildlife and/or human welfare—are considered the largest threat to global biodiversity (Luque *et al.,* 2014). Not all introduced species become ‘invasive’ (Williamson and Fitter, 1996); at a minimum, the introduced species must be physiologically capable of establishing (i.e. surviving and successfully reproducing) and spreading (i.e. range expansion) to ultimately become ‘invasive’ (Blackburn *et al.,* 2011). Invasion science requires a multidisciplinary approach for effective management, and usually evaluates behaviour, ecology, genetics and economics; physiology, however, could be an equally important field to incorporate and is gaining traction as a tool that can contribute to conservation science. Because physiological processes underlie an individual’s response to its new environment (Lennox *et al.,* 2015), some traits could be used as tools to predict invasion success and better inform conservation actions. Assessing the physiological capabilities of invasive fishes has already proven useful in developing and establishing barriers to minimize connectivity and passage (Rahel and McLaughlin, 2018). Yet, most research that has incorporated physiological tools into invasive science research did so to identify traits that can predict species invasiveness (Lennox *et al.,* 2015) and not to inform conservation actions. Given that invasive species impacts will likely be intensified by ongoing climate change (Mainka and Howard, 2010), conservation physiological tools could be pivotal in predicting the extent of potential invasions (Kearney *et al.,* 2009), changing invasive species distributions and managing their ongoing impact in non-native habitats (Madliger *et al.,* 2016).

Which physiological attributes facilitate invasive species establishment and spread?
What physiological characteristics (e.g. metabolic by-products, chemical cues) of invasive species contribute to negative impacts on native species?
In what ways are invasive species behaviourally or physiologically distinct from native ones?
Can physiological vulnerabilities in invasive species be identified and exploited to control them (i.e. know your enemy)?
What is the role of niche construction in the success of biological invasions or adaptation?
How important is phenotypic plasticity (acclimatization capacity) in invasiveness?
How can physiological insights improve the efficacy and impact of biological control plans (e.g. species introductions to control other species)?

### Methods, biomarkers and monitoring

Underpinning conservation physiology is a growing toolbox that includes diverse tools and methods for objectively quantifying the physiological state of organisms (Madliger *et al.,* 2018). These tools need to scale from molecules, cells and organ systems to populations and ecosystems to ensure ecological relevance (Cooke and O’Connor, 2010). Biomarkers sensitive to different stressors and ideally linked to fitness outcomes are needed to generate organism-stressor relationships and to monitor the state of individuals, populations and ecosystems (Bergman *et al.,* 2019). As such, that usually means taking the lab to the field and understanding natural/constitutive vs. stressor-induced changes in a particular biomarker. Doing so requires use of novel and creative technologies such as point-of-care devices (e.g. blood glucose meters that can be used in the field; Stoot *et al.,* 2014) or electronic sensor biologging or biotelemetry tags (Wilson *et al.,* 2015). However, use of new techniques especially in unconventional settings (e.g. measuring hormone levels from respiratory vapour droplets of whales; Hunt *et al.,* 2013) requires validation and assurances that animal welfare is maintained (Putman, 1995). Moreover, even traditional biomarkers such as glucocorticoids (Sheriff *et al.,* 2011; Madliger and Love, 2014) and oxidative stress (Beaulieu and Costantini, 2014) require careful validation when working on new species, new matrices or when trying to contextualize findings (e.g. understand what is baseline, range of responses and physiological consequences). Incorporating physiological biomarkers into routine monitoring programs is still uncommon (Lam and Gray, 2003) but has the potential to revolutionize conservation (Madliger *et al.,* 2018). The research questions outlined here collectively span issues with methods, biomarkers and monitoring and when addressed will ensure that conservation physiologists and the tools they use yield reliable and robust findings that provide meaningful information about the status of organisms, populations and ecosystems.

Which physiological tools/biomarkers used in human clinical care can be ported to conservation physiology, and vice versa?
What are the thresholds of glucocorticoids or other stress-related biomarkers beyond which negative effects on growth, reproduction or survival consistently occur?
Are there physiological measures that can identify or inform tipping points of drought stress in plants, particularly threatened trees and shrubs?
How much have physiological trait-based approaches improved biodiversity forecasting?
What are the hormonal mechanisms underlying behavioural changes in response to environmental change?
What are the physiological ‘early warning signals’ of population decline or collapse?
Can rapidly evolving remote sensing or animal-borne sensor technologies be used to inform conservation of biodiversity?
How can physiological and genetic techniques be used to better characterize the presence and diversity of the microbiome and its ecological relevance?
How can field and laboratory approaches be standardized and ground-truthed across taxa in conservation physiology?
How can physiology be used to better measure, monitor and minimize welfare impacts on wild animals?
What are the most robust physiological biomarkers to assess organismal health and future fitness?
Can broadly applicable models and decision support tools be developed that generalize physiological responses of organisms, scale from individuals to ecosystems and allow us to improve species distribution modelling?
How can the incorporation of physiology into epidemiological modelling increase our ability to predict disease emergence and spread in animal populations?

### Policy, engagement and communication

Conservation policy is ideally based on scientific evidence (Sutherland *et al.,* 2004) and further informed by extensive engagement with relevant stakeholders and rights holders (Reed, 2008). Moreover, policy change depends on political will, which is greatly influenced by public perspectives (Rose *et al.,* 2018). Conservation physiology offers novel insights that readily translate to stakeholders and the public, yet because conservation physiology tends to focus on individuals or below (e.g. genes, cells, organ systems) whereas management efforts tend to focus on populations or ecosystems, there is often a disconnect (Cooke and O’Connor, 2010). Much work is needed to better understand the interface between conservation physiology knowledge and conservation policy and practice (Rose, 2015). Fortunately, there is a growing suite of examples of where physiology has informed conservation practice and policy, although there is also a need to share success stories with a diverse public (Madliger *et al.,* 2016). Moreover, there is a need to enhance engagement and trust between knowledge generators (i.e. conservation physiologists) and knowledge users to garner broader support for conservation physiology research, in line with conservation biology in general (Robinson, 2006; Cook *et al.,* 2013). To date, conservation physiology success stories are rather local in scale (Madliger *et al.,* 2016) but there are certainly opportunities to support more global initiatives such as bending the curve for biodiversity loss or addressing the UN Sustainable Development Goals (Cooke *et al.,* 2020).

Which physiological metrics and endpoints are best suited to informing conservation policy?
What strategies specific to conservation physiology should be used when engaging in knowledge co-production with ‘community scientists’ and other stakeholders and rightsholders?
What strategies can be used to educate and motivate the public to take part (e.g. community science) in conservation physiology research?
In what ways can the value of physiology to conservation be articulated to policymakers and practitioners to increase its upkeep/application (i.e. similar to the success of ecology and conservation genetics)?
What is needed to enable conservation physiology to be adopted and embraced by national and global organizations that guide conservation policy and action?
What are the best ways to make the public aware of and excited about conservation physiology?
How do conservation physiologists best integrate communication into their research programmes to reach diverse audiences including the public, stakeholders and decision-makers?
How can conservation physiology be made a global endeavour that incorporates regional diversity into the discipline
How can conservation physiology enhance action on Sustainable Development Goals (SDGs) and contribute towards a net zero carbon economy?
How can conservation physiology best contribute to the One Health initiative of the World Health Organization that seeks optimal health outcomes recognizing the interconnection between people, animals, plants, and their shared environment?
What are the most effective and compelling ways to incorporate physiology into conservation science courses and encourage a new generation of conservation physiologists?
How can a welcoming, just and inclusive community for aspiring conservation physiologists be created?
What strategic initiatives would help to foster the further development of conservation physiology as a respected, relevant and essential aspect of conservation science and practice?

### Pollution

The panoply of pollutants impacting wildlife is broad and interactive, contributing globally to biodiversity loss (McNeely, 1992; Rawat and Agarwal, 2015). Pollution has evolved from the more obvious types such as air (e.g. burning of fossil fuels) and water (e.g. chemical runoff), to less obvious types including noise, light and microplastic pollution (Cooke *et al.,* 2020). For example, microplastics have been considered a serious threat to freshwater (Lambert and Wagner, 2018), terrestrial (de Souza Machado *et al.,* 2018) and marine (Galloway and Lewis, 2016) systems and the species within them. Only now are efforts underway to understand the potential physiological consequences of microplastics. Because an organism’s physiology often responds rapidly to stressors—including pollutants—evaluating and/or monitoring biomarkers in individuals can offer researchers an opportunity to act before impacts manifest at population or ecosystem levels. Physiological knowledge in itself can be used to identify regulatory thresholds of different pollutants for plants (Das *et al.,* 1997) and animals (Monserrat *et al.,* 2007) and justify actions (e.g. DDT bans after mechanistic action revealed to impact eggs of raptors). Sentinel species have also been widely used since the 19th century (e.g. ‘the canary in the coal mine’) as ecological indicators or biomarkers of pollution (Berthet, 2012). Using physiology to understand how different species are affected by, and respond to, different pollutants, offers an evidence-based tool to develop and implement management actions.

How can physiological biomarkers help us design better barriers to contain noise and light pollution?
How can physiology inform the interactive effects between plastic pollution and climate warming?
What are the physiological responses of animals to heavy metals and other environmental toxicants?
What are the physiological impacts of chemical contaminants on individual physiology, their carry over effects, and how do these effects differ across life stages?
What are the underlying mechanisms mediating biosequestration in trees (to select species for remediation and prevent transfer to other trophic levels)?
What mechanisms are responsible for intra- and inter-specific variation in physiological tolerance of environmental pollutants?
Can physiological monitoring programs quantify the sublethal impacts of pollution and identify areas in most need of recovery efforts?
How do pollutants interfere with physiological systems and hence population dynamics (growth, development, survival)?

### Restoration actions

Recognizing the dramatic effects of humans on the planet, the UN launched the Decade for Ecosystem Restoration in 2019, recognizing that it is not too late to reverse biodiversity decline (Young and Schwartz, 2019). Although conservation physiology is often regarded as a means of identifying problems (e.g. quantifying stress and identifying tolerance thresholds), physiology also has the potential to inform restoration actions (Cooke and Suski, 2008). Ecological restoration implicitly recognizes the interplay of *ex situ* and *in situ* activity contributing to success (Miller *et al.,* 2017; Standards Reference Group SERA, 2017), born out by the engagement of zoos and botanic gardens in ecological restoration and conservation biology (Hardwick *et al.,* 2011; Gilbert *et al.,* 2017). Furthermore, there are synergies between the research questions that underpin ecological restoration (Miller *et al.,* 2017) and those guiding more familiar conservation activities such as reintroductions (Armstrong and Seddon, 2008; Maschinski and Albrecht, 2017). What is less broadly recognized in these fields is the extent to which physiological research can resolve these guiding questions. Conservation physiology can inform all aspects of restoration including optimization of captive breeding programs (e.g. Roth, 2006), seed preservation (Hay and Probert, 2013), reducing stress during translocation (Tarszisz *et al.,* 2014) and identifying organisms (especially plants) that will thrive in a given environment (Ehleringer and Sandquist, 2006). Many resources are devoted to restoration actions yet rarely are they evaluated to assess their effectiveness (Cooke *et al.,* 2019), but there are emerging examples where physiological studies provide key insights (e.g. Bleby *et al.,* 2012; Bateman *et al.,* 2018). To increase the effectiveness of restoration initiatives and ensure that limited resources are invested wisely, conservation physiology has the potential to play an important role in assessing and refining restoration actions.

Which physiological traits best inform selection of taxa for project-specific restoration actions?
Can an understanding of human and domesticated (e.g. livestock) animal physiology or disease processes inform the management of wild or captive animal populations (i.e. nutrition management, disease risk, pollution effects, fertility control)?
Do organisms at the core and edges of populations differ in physiological phenotypes such that this can be exploited to enhance management?
For cases of genetic rescue, assisted migration or translocation, how can physiology help inform rules of practice to avoid inbreeding or outbreeding depression, deleterious breakdown of local adaptation or other hindrances to success?
How can physiological assessments at capture and subsequent monitoring aid in the rehabilitation and release of animals?
What physiological knowledge is needed to further improve the welfare of animals in captivity?
How do land use changes affect critical ecosystem functions involved in restoration successions (e.g. pollinator recruitment, seed germination and emergence)?
How heritable is relevant physiological variation and does it have a role in breeding programs?
If *ex situ* conservation is essential for species recovery, can we specifically ‘harden off’ (i.e. prepare organisms, especially plants, for reintroduction) our reintroduction populations using physiological targets?
In cases of extreme restoration, such as terraforming from ground zero (e.g. mine tailings), is it possible to create physiologically beneficial refuges specific to threatened flora and fauna?
How can candidate species for reintroduction be assessed to ensure that they are physiologically suited to the environment to which they would be introduced?
How does natal vs. adult environmental mismatch influence the success of captive breeding programs?
What is the proportional cost to restoration of collecting and incorporating ecophysiological data in comparison to the costs of suboptimal restoration outcomes?
What non-invasive or minimally invasive physiological tools could be used to improve the success of captive breeding programs?
Which physiological functions in plants can be used to select candidates to restore landscapes following disturbances?
What are the characteristics of environmental refugia that allow them to buffer physiologically and phenotypically diverse communities against global change?

### Threatened species

It is no secret to the conservation research community that much of nature has been lost, and what remains continues to deteriorate. The global rate of species extinction is currently tens to hundreds of times higher than it was in the past 10 million years, largely as a result of anthropogenic activities, and is expected to continue unless drastic actions are taken to reduce drivers (IPBES, 2019). The International Union for Conservation of Nature and Natural Resources (IUCN) uses measurements of population declines as a critical indicator in threat assessments (Mace *et al.,* 2008); actions must be taken to reverse declines, or we are simply monitoring species to their extinction. Physiology can provide valuable causal information underlying such declines by helping identify how individuals acquire, metabolize and allocate resources, and can additionally provide an understanding of how organisms adapt to environmental change (Boersma *et al.,* 2001; Birnie-Gauvin *et al.,* 2017). Many examples exist that describe the benefits of using physiology to support conservation actions, including strategies to protect and restore threatened species populations (see Madliger *et al.,* 2018, for examples). Especially when combined with other techniques (e.g. ecological or behavioural data), conservation physiology can offer tools to evaluate risks and strengthen recovery plans.

Are there physiological similarities between the most vulnerable species and populations of wild organisms?
Can novel intra-specific hybridization programs improve physiological resilience of imperilled species?
How can identifying key physiological requirements of organisms help to locate (model) and prioritize high-quality/high-value habitats?
How can zoos, aquaria and botanical gardens contribute to understanding the physiology and recovery potential of endangered/threatened species?
What physiological data are most useful to IUCN and regional threat assessments to establish the status of populations, species or individuals?
What are the best physiological tools for improving the health, condition and fitness of endangered species?

### Urban systems

Humans have transformed the Earth and nowhere is that more evident than in urban environments. On a global basis, ~55% of the human population lived in urban areas in 2018 and that is anticipated to reach 68% by 2050 according to UN projections (https://www.un.org/development/desa/en/news/population/2018-revision-of-world-urbanization-prospects.html), and the urban landscape is one of the few that are increasing globally (Miller and Hobbs, 2002; Dearborn and Kark, 2010). Urban environments are characterized by extensive landscape modification such as installation of impervious surfaces and development of buildings, and high densities of humans (Rebele, 1994). Nonetheless, urban environments are also ecosystems and are increasingly recognized for their biodiversity structure and function (Savard *et al.,* 2000). These novel ecosystems create opportunities for wildlife (Kowarik, 2011) but there can be negative consequences (Birnie-Gauvin *et al.,* 2016). For example, urban racoons scavenge human trash that can cause physiological alterations in racoons (Schulte-Hostedde *et al.,* 2018). Urban plants may experience continual stress, yet also generate immense benefit to human and wildlife urban dwellers (Calfapietra *et al.,* 2015) while anthropogenic urban disturbances (e.g. noise, light; Sanders *et al.,* 2021) influence the stress physiology of birds (Partecke *et al.,* 2006). Given the increasing urban spread and intensification, there is a need to understand how organisms make a living in these novel ecosystems, why some succeed spectacularly and others fail, and how that knowledge can be exploited to benefit nature and humans.

Could physiological systems that readily/rapidly respond to human disturbance (e.g. heart rate) be used to non-invasively monitor impacts of, and remediation actions at, degraded/urban/disturbed sites?
What is the role of niche construction in the success of biological invasions or adaptation to urban environments?
What are the physiological stressors and niches that are unique to urban ecosystems?
What methods best predict which species will respond well to urban environments on the basis of physiological traits, compared with those that do poorly, regardless of their rarity in natural ecosystems?
Is there a physiological basis to why the responses of many species to urbanization and other anthropogenic land-use changes differ biogeographically?
Can physiology be used to identify opportunities for creating and refining urban green spaces and infrastructure (e.g. green roofs) that create maximal benefits for both wildlife and humans?

## Synthesis and conclusion

Because conservation physiology is a nascent discipline, many questions remain to be tackled by the research community. A diverse group of conservation physiology researchers generated a list of research questions to guide the further development of this field so that we can deliver relevant science to inform the conservation and management of biodiversity and natural resources (*sensu*  Rudd, 2011). The evolution of conservation physiology as a relevant and practical conservation discipline depends on close engagement with, and accessibility to, managers, decision-makers and policy makers. These questions offer researchers an opportunity to work with other actors such as policy makers and practitioners to develop practical pathways for integrating physiological tools and approaches into traditional conservation approaches.

The research themes identified here are diverse and reflect the enormous scope for conservation physiology to contribute to conservation and biodiversity protection. We addressed questions relating to methodological approaches, threat assessments, restoration interventions and policy and communication. Although conservation physiology is inherently an applied discipline (Cooke *et al.,* 2013), it is underpinned by fundamental research and theory and so we also included questions related to physiological variation, adaptation and evolution. Many of the questions are sufficiently broad in scope that they can serve as the basis for dissertations and career-spanning research programs; some of the questions are relatively easy to address while others are complex. Some questions require experimentation and field or laboratory work while others can be advanced or addressed through modelling or evidence synthesis activities (e.g. meta-analysis). We recognize that science, particularly when addressing conservation and management, is often context-dependent and we therefore attempted to limit questions that were specific to an ecosystem or taxon. In almost all instances (not just in conservation physiology but in science more generally), there is no single study or experiment that will satisfactorily answer a question given the context specificity of most conservation physiology research, which spans ecosystems, taxa and biological scales (Tomlinson *et al.,* 2018). This is not ‘stamp collecting’ but rather a reality of the inherent complexity of both conservation problems and their solutions. In fact, it is a diverse and comprehensive literature base that enables evidence synthesis and evidence-based decision-making (Cooke *et al.,* 2017). Conservation physiology offers a wealth of opportunities to integrate tools and approaches that span all levels of biological organization, that brings together and incorporates other research fields and that can be tailored to suit specific problems, populations, taxa or management requirements.

To address the questions presented here will require the coordinated efforts of established researchers and conservation practitioners; however, these questions are presented in recognition that many of the conservation problems of today and tomorrow will be solved by the next generation of scientists (Jeanson *et al.,* 2020). We are convinced that the questions shared here will inspire future scientists and provide them with meaningful ways that they can contribute to the development of conservation physiology and, more importantly, to enhance its impact. There are many ways that aspiring conservation physiologists can engage in pressing issues facing the world today (see horizon scan in Cooke *et al.,* 2020) and the questions presented here provide an opportunity to do so. Because the questions generated here were contributed by researchers, future exercises would benefit from inclusion of various knowledge users like policy makers and practitioners. In doing so, opportunities would be created to contrast the research questions of importance here to knowledge generators and knowledge users (Rudd and Fleishman, 2014). As conservation physiology matures and increases the diverse ways in which it serves the conservation science community, we anticipate that collective efforts devoted to tackling the questions outlined here will yield benefits for biodiversity and humanity.
